# The relationship of changes in insulin demand and insulin adequacy over the life course

**DOI:** 10.1007/s00125-024-06328-9

**Published:** 2024-11-29

**Authors:** Yingchai Zhang, Claudia H. T. Tam, Eric S. H. Lau, Noel Y. H. Ng, Aimin Yang, Baoqi Fan, Hongjiang Wu, Cadmon K. P. Lim, Elaine Y. K. Chow, Andrea O. Y. Luk, Alice P. S. Kong, Wing Hung Tam, Juliana C. N. Chan, Ronald C. W. Ma

**Affiliations:** 1 Department of Medicine and Therapeutics, Prince of Wales Hospital, The Chinese University of Hong Kong, Hong Kong, China; 2 Hong Kong Institute of Diabetes and Obesity, Prince of Wales Hospital, The Chinese University of Hong Kong, Hong Kong, China; 3 Li Ka Shing Institute of Health Sciences, Prince of Wales Hospital, The Chinese University of Hong Kong, Hong Kong, China; 4 Department of Obstetrics and Gynaecology, Prince of Wales Hospital, The Chinese University of Hong Kong, Prince of Wales Hospital, Hong Kong, China; 5 CUHK Medical Centre, Shatin, Hong Kong, China

**Keywords:** Insulin adequacy, Insulin demand, Insulin secretion, Insulin sensitivity, Life course, Natural logarithm of ratio between the exponential functions of insulin adequacy and insulin demand, Standardised major axis regression

## Abstract

**Aims/hypothesis:**

Insulin requirements in the human body undergo continuous changes in response to growth and development. We assessed the life course relationships between insulin demand and insulin adequacy.

**Methods:**

Three independent Chinese cohorts (204 children, aged [mean ± SD] 7.0 ± 0.5 years; 214 adolescents, aged 15.0 ± 1.8 years; 605 adults, aged 41.5 ± 9.3 years), recruited between 1998 and 2013, underwent OGTT tests. Indices of insulin sensitivity and insulin secretion were calculated based on paired glucose/insulin values during fasting, early phase and late phase of OGTT. Insulin demand and insulin adequacy were calculated by standardised major axis (SMA) regression from the paired insulin sensitivity and secretion indices. We derived the natural logarithm of ratio between the exponential functions of insulin adequacy and insulin demand (RAD) index for further evaluating the relationship between insulin demand and adequacy. The risk of abnormal glucose tolerance (AGT) was evaluated by logistic regression analyses. Area under the receiver-operating characteristic curve (AUC-ROC) analyses, net reclassification improvement (NRI) and integrated discrimination improvement (IDI) indices were used to demonstrate the discriminative value of the RAD method model.

**Results:**

Adolescents had the lowest insulin sensitivity and the highest insulin secretion in all phases (fasting, early and late phase) of the OGTT, as compared with children and adults in each phase (all *p*<0.001). Adolescents had the highest insulin demand in all phases and lowest insulin adequacy in the fasting phase (*p*<0.001). In general, adults had the lowest insulin adequacy in both the early phase (*p*>0.05) and late phase (*p*<0.001) of the OGTT. Adolescents had negative RAD values irrespective of overweight and obesity, while, in general, children and adults had positive RAD values (*p*<0.001 between age groups in each of the fasting, early and late phases of the OGTT). Participants with RAD values below the 25th percentile had a higher risk of AGT compared with those above the 25th percentile (fasting-phase OR 1.86 [95% CI 1.18, 2.91]; early-phase OR 1.99 [95% CI 1.24, 3.19]; late-phase OR 2.49 [95% CI 1.57, 3.97]). The late-phase RAD index had the best performance in evaluating the risk of AGT compared with the fasting- and early-phase RAD indices (late-phase AUC-ROC = 0.635 [95% CI 0.583, 0.687]; late-phase NRI = 0.350 [95% CI 0.190, 0.510]; late-phase IDI = 0.033 [95% CI 0.015, 0.050]).

**Conclusions/interpretation:**

The relationship between insulin demand and insulin adequacy changed throughout the life course. Adolescents had an imbalanced relationship between insulin demand and insulin adequacy, while, in general, children and adults had a balanced relationship. RAD is a novel index that was used to efficiently describe this relationship and evaluate the risk of AGT.

**Graphical Abstract:**

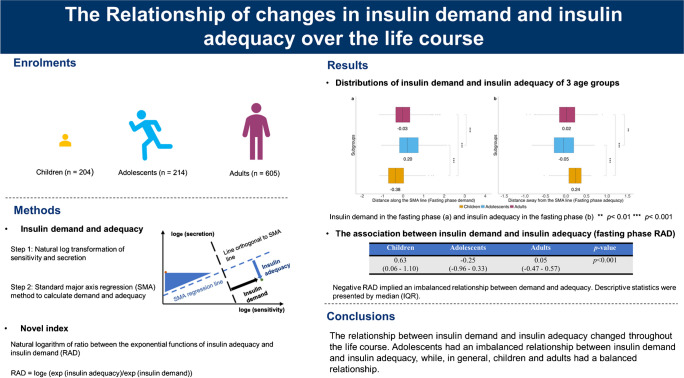

**Supplementary Information:**

The online version contains peer-reviewed but unedited supplementary material available at 10.1007/s00125-024-06328-9.


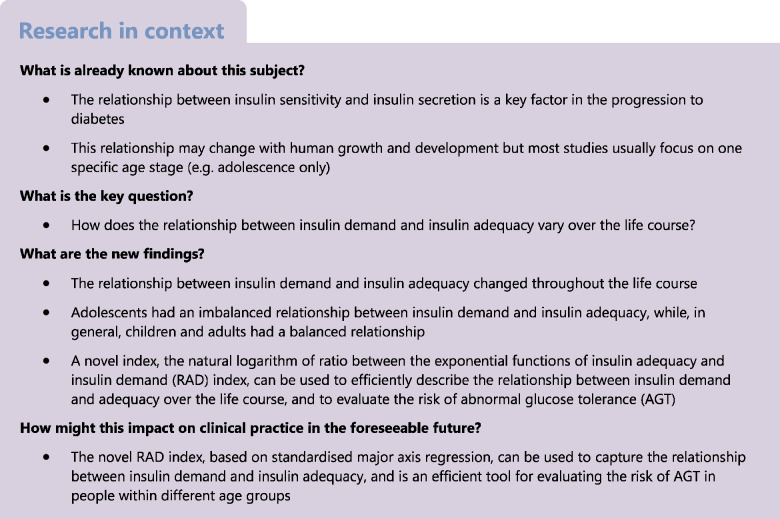


## Introduction

A balanced relationship between insulin sensitivity and insulin secretion is critical for maintaining normal glucose metabolism [1]. In general, physiologically, a decrease in insulin sensitivity is accompanied by a compensatory increase in insulin secretion and vice versa [2]. The relationship between insulin sensitivity and insulin secretion may undergo changes at specific life stages [3–5]. So far, most studies have mainly investigated the relationship between insulin sensitivity and secretion at specific periods of the life course, such as from early to late puberty or from adolescence to young adulthood [5, 6], as opposed to over the life course. In addition, the differences in the relationship between insulin secretion and insulin sensitivity after glucose ingestion, in both the early and late phase of an OGTT, is often overlooked [7, 8].

The standardised major axis (SMA) regression method [9], a precise bivariate line-fitting method minimising the residuals of both bivariate variables, has recently been utilised in the field of insulin sensitivity and secretion to evaluate the risk of development of type 2 diabetes [10]. The SMA method minimises residuals of both insulin sensitivity (residuals in sensitivity direction, representing insulin demand) and insulin secretion (residuals in secretion direction, representing insulin adequacy), providing an accurate estimation of the relationship between insulin demand and insulin adequacy [10].

In the current study, we aimed to use the SMA regression method to evaluate changes in the relationship between insulin demand and adequacy over the life course.

## Methods

### Data source and participants

This study included Chinese participants from Hong Kong over the life course (in childhood [5–9 years old], adolescence [10–19 years old] and adulthood [≥20 years old]) with an age range of 5.5 years to 73.0 years, recruited between 1998 and 2013 (electronic supplementary material [ESM] Methods). Ethnicity (Chinese) and sex were self-reported. Additional details regarding the inclusion and exclusion criteria are provided in ESM Fig. 1 and ESM Methods. Informed consents were obtained from adult participants and assent was obtained from parents of children involved in the study. Ethical approval for this study was obtained from the Joint Chinese University of Hong Kong-New Territories East Cluster Clinical Research Ethics Committee.

### Definitions and equations

We defined glucose tolerance using definitions of the American Diabetes Association based on OGTT tests (ESM Methods). Participants who self-reported having diabetes or received treatments for diabetes, or with a diagnosis of diabetes as from OGTT, were excluded from the analysis. Abnormal glucose tolerance (AGT) was defined as one of the following conditions: (1) isolated impaired fasting glucose (i-IFG); (2) isolated impaired glucose tolerance (i-IGT); (3) a combination of impaired fasting glucose and impaired glucose tolerance (IFG+IGT); and (4) diabetes.

Indices of insulin sensitivity and insulin secretion were derived from paired plasma glucose–insulin measurements during three phases of the OGTT: the fasting phase, early phase and late phase [11–13]. Phases were based on physiological responses of insulin before or after glucose ingestion. Insulin sensitivity and insulin secretion during the fasting phase were expressed as HOMA2-%S and HOMA2-%B, respectively, estimated using the online HOMA2 calculator v2.2.3 (www.dtu.ox.ac.uk/homacalculator/). The reciprocal of hepatic insulin sensitivity index (1/HISI) was used to assess insulin sensitivity during the early phase of the OGTT. The calculation of 1/HISI involved multiplying the AUCs of glucose and insulin during 0–30 min of the OGTT and then determining the reciprocal of the product [14]. The Stumvoll first-phase index (Stumvoll index-1) was used to evaluate first-phase insulin secretion [12, 14]. Stumvoll index-1 was calculated using the following equation: 1194 + 4.724 × fasting insulin − 117.0 × glucose at 60 min + 1.414 × insulin at 60 min. Insulin sensitivity and insulin secretion in the late phase of the OGTT were calculated using the Matsuda index and Stumvoll second-phase index (Stumvoll index-2) [11–13]. To optimise the accuracy of insulin sensitivity assessment, we integrated glucose/insulin values at all five timepoints using the trapezoidal method to calculate the Matsuda index, which was highly correlated (*r*=0.98, *p*<0.001) with the common method which uses only four timepoints (0 min, 30 min, 60 min and 120 min). The Stumvoll index-2 was calculated using the equation: 295 + 0.349 × insulin at 60 min − 25.72 × glucose at 60 min + 1.107 × fasting insulin. The units for glucose and insulin used for these calculations were consistent with the original articles or websites cited above [3, 11–14].

In this study, we used the SMA regression method to estimate the values of insulin demand and insulin adequacy (ESM Fig. 2). The centroid point is defined by the mean of the natural logarithm values of insulin sensitivity and insulin secretion. For a particular coordinate, insulin demand is defined as distance along the SMA regression line and insulin adequacy as the perpendicular distance away from the SMA regression line. To investigate the balance between insulin demand and adequacy, we developed the natural logarithm of ratio between the exponential functions of insulin adequacy and insulin demand (RAD) index, calculated as (where exp stands for exponential function):

$$\text{RAD}={\text{log}}_e\left(\frac{\text{exp}\left(\mathrm{insulin\;adequacy}\right)}{\text{exp}\left(\mathrm{insulin\;demand}\right)}\right)$$

The RAD index amplifies the ‘mismatch’ between insulin demand and insulin adequacy, and precisely measures the balance between demand and adequacy.

The age- and sex-specific BMI cutoffs used to define overweight/obesity are provided in the ESM Methods. Blood pressure was measured using a calibrated electronic device (Omron 705; Omron Corporation, Japan) after participants were sitting for 5 min. The mean value of three blood pressure measurements was used for final analyses. Venous blood samples, collected at baseline, were used for glucose, insulin and lipid profile (cholesterol, plasma triglycerides, HDL-cholesterol and LDL-cholesterol) tests (ESM Methods).

### Statistical analyses

Baseline characteristics were presented as mean ± SD, median (IQR) or *n* (%) as appropriate. One-way ANOVA tests for normally distributed data or Kruskal–Wallis tests for non-parametric analyses were used to compare differences among different age groups, as appropriate. The Bonferroni correction method was used to adjust for post hoc multiple comparisons. *Χ*^2^ tests were employed for comparisons between categorical variables.

Logistic regression was used to estimate the ORs and 95% CIs for dichotomous outcomes to evaluate the risk of AGT/diabetes. We examined the discriminative value of insulin demand–adequacy indices or the derived RAD indices in three phases using area under the receiver-operating characteristic curve (AUC-ROC) analysis. We used net reclassification improvement (NRI) and integrated discrimination improvement (IDI) indices to demonstrate the improved efficacy of the insulin demand–adequacy/RAD models, as compared with the base model, which only included age and sex. The insulin demand–adequacy method refers to a model that included insulin demand and insulin adequacy indices, while the RAD method refers to a model that included RAD indices. For both methods, models were adjusted for age and sex.

Statistical significance was defined as a two-sided *p* value<0.05, unless otherwise specified. For post hoc comparisons, a two-sided *p* value<0.0167 was considered significant for each of the two-group comparisons among the three age groups. All statistical analyses were performed using R version 4.2.2 software (www.r-project.org/).

## Results

### Characteristics of participants at baseline

A total of 204 children, 214 adolescents and 605 adults from three independent prospective cohorts were included to form the life course cohort at baseline (ESM Table 1). The proportion of male participants ranged from 43.5% to 53.4% among the three age groups, with no statistical difference in the proportion of male participants. The mean ± SD age of the children, adolescents and adults were 7.0 ± 0.5 years, 15.0 ± 1.8 years and 41.5 ± 9.3 years, respectively. Adults (including those who were not overweight or obese) had higher BMI than children and adolescents. Glucose levels were highest in adults and lowest in children at 0 min and 120 min. During the OGTT, we observed a sharp decrease in glucose levels in children between 30 min and 120 min (ESM Fig. 3). In adolescents and adults, we observed a delay in this decrease, which occurred at 60 min. Adolescents had the highest insulin levels during the whole OGTT period, compared with the other two age groups (ESM Fig. 3). At baseline, the proportion of individuals with AGT (not including diabetes) was highest in adults, followed by adolescents and then children, with the proportion being 22.0%, 15.9% and 3.4% respectively (ESM Table 1).

### Insulin sensitivity and secretion over the life course

**Fig. 1:**
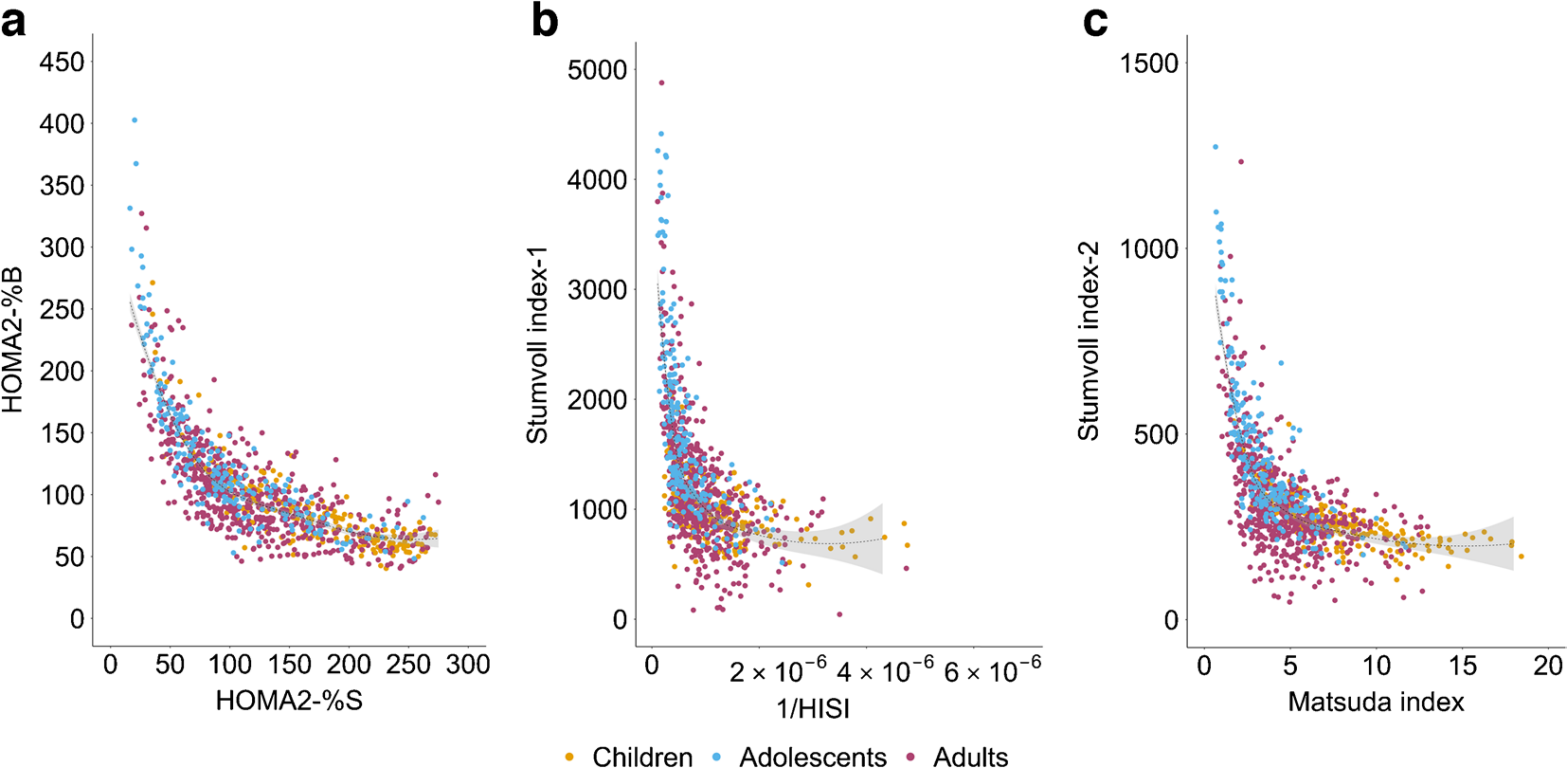
Insulin sensitivity (*x* axis) and secretion indices (*y* axis) over the life course (in children, adolescents and adults) during the fasting, early and late phase of the OGTTs. (**a**) Fasting phase insulin sensitivity–insulin secretion indices, presented by HOMA2-%S and HOMA2-%B, respectively. (**b**) Early-phase insulin sensitivity–insulin secretion indices, presented by 1/HISI and the Stumvoll index-1, respectively. (**c**) Late phase insulin sensitivity–insulin secretion indices, presented by the Matsuda index and Stumvoll index-2, respectively. The fitted curvilinear line of insulin sensitivity and insulin secretion (black dotted lines) and the 95% CI of the curvilinear line (grey-shaded area) are also shown on each graph

When analysed as a life course cohort, there was a curvilinear relationship between insulin sensitivity and insulin secretion during the three phases of the OGTT, with individuals at the extremes of the curvilinear line being prone to metabolic decompensation due to small changes in either of the indices relating to insulin sensitivity or insulin secretion (Fig. 1). The data points for adults (with insulin sensitivity plotted on the *x* axis and insulin secretion on the *y* axis) were clustered around the middle of the curvilinear line, while those for adolescents and children were clustered at the upper and lower ends respectively. When comparing the median values of these indices across the age stages of the life course, adolescents had the highest insulin secretion but the lowest insulin sensitivity, as compared with children and adults for all three phases of the OGTT (ESM Table 2; *p*<0.001), even when stratifying for those with normal glucose tolerance (NGT) and/or BMI not in the overweight/obese range (ESM Table 3).

When comparing among the three OGTT phases, the coordinates for all three age groups were distributed relatively centrally around the curvilinear line in the fasting phase, with a few outlying data points mainly for adolescents (Fig. 1). In both the early and late phases, the coordinates were observed to exhibit greater dispersions in the insulin sensitivity direction than those in the fasting phase, with more data points at the two extreme ends. High secretion dispersions were mainly found in adolescents at the higher extreme ends and low secretion dispersions were mainly found in adults at the lower extreme ends. When limited to participants with NGT and/or without overweight/obesity, we observed a reduction in the number of outlying points, but the dispersion patterns in the three life course stages remained consistent with the results above (ESM Fig. 4).

### Insulin demand and insulin adequacy over the life course

**Fig. 2:**
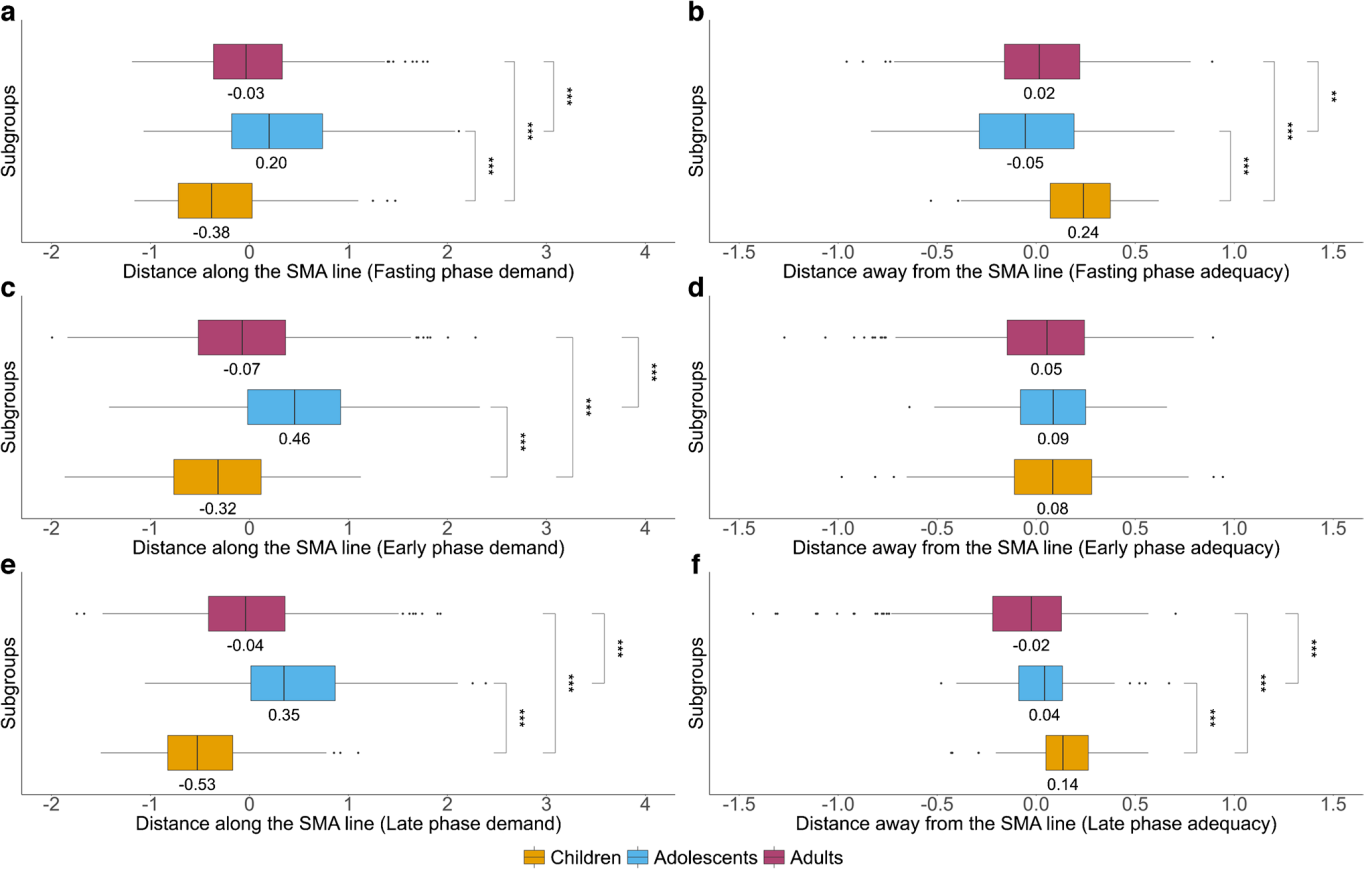
Insulin demand and insulin adequacy over the life course (in children, adolescents and adults). (**a**–**f**) Insulin demand (**a**, **c**, **e**) and insulin adequacy (**b**, **d**, **f**) were derived from insulin sensitivity and insulin secretion indices in the fasting (**a**, **b**), early (**c**, **d**) and late (**e**, **f**) phase of the OGTT using the SMA regression method. Insulin sensitivity in the fasting phase, early phase and late phase of the OGTT was represented by HOMA2-%S, 1/HISI and the Matsuda index, respectively. Insulin secretion in the fasting phase, early phase and late phase of the OGTT was represented by HOMA2-%B, the Stumvoll index-1 and the Stumvoll index-2, respectively. The middle lines in the box plots show the median values for each age group, while the upper lines and lower lines show the first quartiles (Q1) and the third quartiles (Q3), respectively. Lower outliers indicate values less than Q1−(1.5×IQR) and higher outliers indicate values greater than Q3+(1.5×IQR). **p<0.01, ***p<0.001

We applied the SMA regression method to evaluate the relationship between insulin demand (distance along the SMA regression line) and insulin adequacy (distance away from the SMA regression line) (ESM Fig. 2). In all phases of the OGTT, adolescents had the highest insulin demand, followed by adults and then children (Fig. 2; *p*<0.001 for all age group comparisons in all OGTT stages). For insulin adequacy, adolescents had the lowest value in the fasting phase of the OGTT (*p*<0.01), while adults had the lowest values in both early and late phases, with statistical significance only in the late phase (*p<*0.001). In general, these trends remained consistent in participants with NGT and/or without overweight/obesity (ESM Fig. 5).

The novel RAD index was used for further investigations of the relationship between insulin demand and insulin adequacy. Adolescents had the lowest RAD values in all three phases, compared with children and adults (Table 1), and these were all negative, indicating an imbalanced insulin demand–adequacy relationship. This trend was consistent even in adolescents without AGT and/or overweight/obesity (ESM Table 4). In children and adults, the insulin demand was generally balanced by the insulin adequacy response, as indicated by positive RAD values (Table 1). Although a slightly negative RAD value was observed in adults in the late phase of the OGTT (Table 1), RAD values were all positive when stratified by NGT and/or non-obese/non-overweight participants (ESM Table 4; *p*<0.001 between age groups in each of the fasting, early and late phases of the OGTT).

**Table 1:** RAD values of the life course cohort during three phases of the OGTT

Variables	Children	Adolescents	Adults	*p* value
*n*	204	214	605	
Fasting-phase RAD^a^	0.63 (0.06 – 1.10)	−0.25 (−0.96 – 0.33)	0.05 (−0.47 – 0.57)	<0.001
Early-phase RAD^a^	0.40 (−0.23 – 0.97)	−0.44 (−0.94 – 0.14)	0.13 (−0.42 – 0.62)	<0.001
Late-phase RAD^a^	0.67 (0.26 – 1.02)	−0.34 (−0.85 – 0.09)	−0.04 (−0.49 – 0.39)	<0.001

Descriptive statistics are presented as median (IQR)

RAD was calculated using the following formula, based on insulin demand and insulin adequacy during different OGTT phases: $\text{RAD}={\text{log}}_e\left(\frac{\text{exp}\left(\mathrm{insulin\;adequacy}\right)}{\text{exp}\left(\mathrm{insulin\;demand}\right)}\right)$

^a^Statistical differences were observed between two groups (children vs adolescents, children vs adults, and adults vs adolescents) using post hoc tests

### Risk of AGT and diabetes assessed using the insulin demand–adequacy method and RAD method

Among 204 children, 115 children had both baseline and follow-up records for glucose tolerance. Among these children, 87 children had NGT, 19 children had i-IFG, five children had i-IGT, four children had IFG+IGT and no children had diabetes at follow-up. In the adult group, 359 of 605 individuals had glucose tolerance records at both baseline and follow-up. Of these adults, 214 adults had NGT, 25 adults had i-IFG, 37 adults had i-IGT, 14 adults had IFG+IGT and 69 adults had diabetes at follow-up. The adolescent group only had fasting plasma glucose data at follow-up, which was not sufficient for defining glycaemic status and, hence, this group was excluded from the analyses of prospective evaluation of glucose tolerance. We compared the baseline characteristics of children and adults with and without follow-up glucose tolerance records and, for the majority of variables, there was no statistically significant difference (ESM Table 5).

We evaluated the risk of AGT (i-IGT, i-IFG, IFG+IGT and diabetes) using both the insulin demand–adequacy method and the RAD method based on follow-up glucose tolerance records. For the insulin demand–adequacy method, we stratified all participants into four groups based on the median values of insulin demand and insulin adequacy at baseline: low demand–high adequacy (LD–HA), high demand–high adequacy (HD–HA), high demand–low adequacy (HD–LA) and low demand–low adequacy (LD–LA). The insulin demand–adequacy distributions of participants are shown in ESM Fig. 6a–c for children and adults. We considered the HD–LA group as the at-risk group, due to having the highest insulin demand but lowest insulin adequacy, and combined the LD–HA, HD–HA, and LD–LA groups as the reference group (Table 2). To keep the sample size of participants in the at-risk group relatively consistent with the insulin demand–adequacy method, we considered participants with RAD values below the 25th percentile as the at-risk group when using the RAD method, and regarded those above the 25th percentile as the reference group (Table 2). The findings showed that the HD–LA group had a higher risk of AGT compared with the reference group when assessed using the insulin demand–adequacy method, with statistical significance only in the fasting and late phase of the OGTT (fasting-phase OR=1.55 [95% CI 1.04, 2.30]; early-phase OR=1.35 [95% CI 0.88, 2.04]; late-phase OR=2.62 [95% CI 1.72, 4.02]). Accordingly, using the RAD method, participants with RAD values below the 25th percentile had a higher risk of AGT compared with those with RAD values above the 25th percentile, with statistical significance in all three phases of the OGTT (fasting-phase OR=1.86 [95% CI 1.18, 2.91]; early-phase OR=1.99 [95% CI 1.24, 3.19]; late-phase OR=2.49 [95% CI 1.57, 3.97]). The base model to evaluate the risk of AGT was adjusted for age and sex, with an AUC-ROC value of 0.578.

**Table 2:** Performance of the insulin demand–adequacy method and the RAD method in evaluating the risk of AGT

Methods	Fasting phase	Early phase	Late phase
Insulin demand–adequacy method^a^			
OR (95% CI)	1.55 (1.04, 2.30)	1.35 (0.88, 2.04)	2.62 (1.72, 4.02)
AUC-ROC (95% CI)	0.607 (0.554, 0.659)	0.589 (0.537, 0.642)	0.645 (0.593, 0.696)
NRI (95% CI)	0.256 (0.075, 0.436)	0.131 (−0.039, 0.301)	0.445 (0.273, 0.618)
IDI (95% CI)	0.010 (0.001, 0.019)	0.004 (−0.002, 0.010)	0.043 (0.024, 0.063)
RAD method^b^			
OR (95% CI)	1.86 (1.18, 2.91)	1.99 (1.24, 3.19)	2.49 (1.57, 3.97)
AUC-ROC (95% CI)	0.613 (0.560, 0.665)	0.605 (0.553, 0.657)	0.635 (0.583, 0.687)
NRI (95% CI)	0.256 (0.095, 0.417)	0.233 (0.078, 0.387)	0.350 (0.190, 0.510)
IDI (95% CI)	0.016 (0.004, 0.028)	0.017 (0.005, 0.030)	0.033 (0.015, 0.050)

The base model was adjusted for age and sex (AUC-ROC=0.578). The insulin demand–adequacy models and RAD models were also adjusted for age and sex

^a^In the insulin demand–adequacy method, participants were divided into four groups stratified by the median values of insulin demand and insulin adequacy: LD–HA, HD–HA, HD–LA and LD–LA. The LD–LA+HD–HA+LD–HA combined group was taken as the reference group for comparisons with the at-risk group (the HD–LA group)

^b^In the RAD method, participants were divided into four groups according to quartiles of RAD values, with individuals with RAD values above the 25th percentile being the reference group, for the comparisons with the at-risk group (individuals with RAD values below the 25th percentile)

We used the insulin demand–adequacy method and the RAD method models to evaluate the risk of AGT in the three OGTT phases. We observed the highest AUC-ROC, NRI and IDI values in the late phase of the OGTT, compared with the fasting phase and the early phase, using both methods (Table 2). When comparing between the two methods, the RAD method was more stable and efficient than the insulin demand–adequacy method as the NRI and IDI values were all significant in the three OGTT phases, while the NRI and IDI values derived using the insulin demand–adequacy method were non-significant in the early phase of the OGTT (Table 2). We built a glycaemic trajectory by selecting adult participants with NGT at baseline and then comparing the baseline RAD values according to glycaemic status at the follow-up visit. The NGT at baseline–NGT at follow-up group had higher RAD values than the NGT at baseline–AGT at follow-up group in all three phases of the OGTT, with differences between groups being statistically significant in the early phase and late phase of the OGTT (ESM Table 6), which was in accordance with the RAD results above.

For sensitivity analyses, we used both the insulin demand–adequacy method and the RAD method to evaluate the risk of diabetes in adults (only the adult group had adequate numbers of incident diabetes diagnoses at follow-up). The insulin demand–adequacy distributions for adults are shown in ESM Fig. 6d–f. Both the HD–LA group and the group with RAD values below the 25th percentile had a higher risk of diabetes compared with their respective reference groups when using the insulin demand–adequacy method and the RAD method, respectively (ESM Table 7). The base model to evaluate the risk of diabetes was adjusted for age and sex, with an AUC-ROC value of 0.541. Consistent with our findings for AGT risk, both the insulin demand–adequacy method and the RAD method performed best for evaluating the risk of diabetes in the late phase of the OGTT, as compared with the other two OGTT phases, and the RAD method was more stable and efficient than the insulin demand–adequacy method (ESM Table 7).

## Discussion

In this study, we examined the insulin demand–insulin adequacy relationship during growth and developmental stages of the life course. Adolescents had the lowest insulin sensitivity and the highest insulin secretion compared with children and adults across all three phases of the OGTT. Our results showed that adolescents had the highest demand in all three OGTT phases and the lowest insulin adequacy in the fasting phase of the OGTT. Adults had the lowest adequacy in both early and late phases of the OGTT in general (although differences between age groups were not significant for the early phase). Our novel index, RAD, further explained the relationship between insulin demand and insulin adequacy. In individuals without diabetes, we observed a generally balanced relationship between insulin demand and adequacy in children and adults but an imbalanced relationship in adolescents. Compared with the insulin demand–adequacy method, the RAD method was more stable and consistent in evaluating the risk of incident AGT and diabetes.

The balance between insulin sensitivity and insulin secretion is affected by hormone production during the life course. In our life course cohort, which included individuals who were free of diabetes at baseline, adolescent participants showed the lowest level of insulin sensitivity but had the highest level of insulin secretion compared with their counterparts in childhood and adulthood. This phenomenon is likely attributable to the metabolic and hormonal changes that occur with growth and development over the life course [15–18]. Growth hormone (GH) and sex steroids have relatively low production in the pre-pubertal childhood stage. When a growth spurt occurs in pubertal adolescents, the hypothalamic–pituitary–gonadal axis becomes highly activated, which triggers a cascade of events. The increased amplitudes of pulsatile secretion of follicle stimulating hormone and luteinising hormone, followed by a marked elevation of gonadal sex steroids, enhances GH and IGF-1 production [15]. GH has direct effects on glucose metabolism by increasing hepatic gluconeogenesis and adipocyte lipolysis, as well as decreasing peripheral glucose uptake, which contributes to lower insulin sensitivity [19]. Similarly, IGF-1, the mediator of GH, has also been reported to be associated with insulin resistance, even at low levels [20]. After peaking in puberty, both GH and IGF-1 gradually decline as individuals move towards adulthood [18], which also supports the observed reverted impairment in insulin sensitivity in our adult participants.

The relationship between insulin sensitivity and insulin secretion is pivotal in the progression to AGT/diabetes [21]; however, there is a strong need for efficient tools to evaluate this relationship. We calculated insulin demand and insulin adequacy by the SMA regression using paired insulin sensitivity and insulin secretion indices. Furthermore, we measured this relationship using a single index, our novel RAD index, instead of the usual method of using two paired indices. RAD was an efficient tool that demonstrated an imbalanced relationship between insulin demand and insulin adequacy in adolescents and a balanced relationship in children and adults, simply indicated by positive or negative values (a positive RAD indicates a balanced relationship between insulin demand and insulin adequacy, while a negative RAD indicates an imbalanced relationship). Compared with the paired insulin demand–adequacy method, the single RAD method also demonstrated efficiency and stability in evaluating the risk of AGT, with statistically significant results in all three OGTT phases. Youth-onset type 2 diabetes is considered to have a more aggressive disease course compared with adult-onset type 2 diabetes, and is associated with increased risk of severe complications and comorbidities [22, 23]. According to the TODAY study, in individuals with young-onset type 2 diabetes, the cumulative incidence of any microvascular complication was 50% by 9 years and 80% by 15 years of diabetes duration, corresponding to a 60% prevalence of having any microvascular complication approximately 13 years after diagnosis [23]. Given its efficiency and stability, the RAD index has the potential to be a valuable tool for evaluating the risk of young-onset type 2 diabetes in clinical evaluation, especially in early detection of type 2 diabetes during or after puberty, a period marked by rapid growth. In the EarlyBird study, researchers reported a progressive increase in severity of insulin resistance in children who were around 7 years old, 3–4 years before they reached Tanner Stage 2, or when they had increases in detectable luteinising hormone marking the beginning of puberty [24]. Rising adiposity and IGF-1 together explained 34% of the variance in insulin resistance in boys and 35% in girls over the 3 years preceding pubertal onset. Given the rising epidemic of young-onset diabetes and its health implications [25], our results highlight the significance of the adolescence period, which is characterised by an insulin demand–insulin adequacy imbalance during puberty. This potentially imbalanced relationship between insulin demand and insulin adequacy may easily decompensate in the presence of concomitant weight gain or obesity.

Our study has several strengths. First, we combined three independent cohorts at different stages along the life course to examine the insulin demand–adequacy relationship using RAD indices of insulin sensitivity and secretion. This differs from other studies that only focused on specific stages of the life course. Second, we confirmed the potential utility of the RAD methods in evaluating insulin demand/adequacy and the risk of AGT for research and clinical practice, especially for young-onset diabetes. Our study also has limitations. First, the life course analyses were derived using three independent cohorts with different age groups rather than prospective analyses of the same individuals. Admittedly, it is impractical to follow individuals from birth to senior age in population-based studies. Besides, our method aligned with recommendations of combining longitudinal data from different cohorts to examine the life course trajectory [26]. Second, we did not have sufficient data to define glucose tolerance in the adolescent group during follow-up and did not have data on Tanner Stage and puberty-related hormones to define the onset of puberty. In this study we assumed the children were pre-pubertal while adolescents were in Tanner Stage 4/5 based on the growth charts for Hong Kong children (available from www.dh.gov.hk/english/useful/useful_HP_Growth_Chart/useful_HP_Growth_Chart.html; accessed 28 September 2024). Third, we evaluated insulin sensitivity–insulin secretion and insulin demand–insulin adequacy relationships using data from OGTT tests instead of the gold standards of clamp tests or frequently-sampled IVGTT (FSIVGTT). Nonetheless, we optimised our model by including multiple timepoints during the three phases of the OGTT, and excluded participants with chronic kidney diseases, which could affect the results. We acknowledge that calculations of insulin sensitivity and secretion covering 0–120 min of the OGTT may be partially influenced and confounded by fasting measurements, and that dividing the OGTT into three phases without using exact timepoints is somewhat arbitrary. Some potential factors such as liver lipid content [27], GH [28], carbohydrate and energy availability [29], ethnicity [30] and puberty may also have effects on hepatic glucose clearance and, subsequently, affect insulin sensitivity and secretion. Fourth, in our models for the risk of AGT and diabetes, we only adjusted for age and sex without considering other covariates, such as family history of diabetes and history of cardiovascular diseases. Our models were merely based on glucose and insulin measurements and, thus, may have lower predictive values than models with more advanced biomarkers or those that incorporate indicators of glucose tolerance. Finally, the children included in this study were offspring of mothers participating in the HAPO study [31, 32], which had a primary aim to investigate hyperglycaemia in pregnancy and adverse pregnancy outcomes. Consequently, we only had information on maternal gestational diabetes and not on the paternal history of diabetes. In these children, the occurrence of cardiovascular diseases was very rare and, hence, cannot be used as a predictor for diabetes.

### Conclusions

In conclusion, our study demonstrated that the relationships between insulin sensitivity and insulin secretion, and the relationships between insulin demand and insulin adequacy underwent changes throughout the life course. Adolescents had an imbalanced relationship between insulin demand and insulin adequacy, while, in general, children and adults had a balanced relationship. RAD was a novel index that was used to efficiently describe the relationship between insulin demand and adequacy and evaluate the risk of AGT.

## Supplementary Information

Below is the link to the electronic supplementary material.

ESM (PDF 1827 KB)

## Data Availability

Data are available from the corresponding author upon reasonable request.

## Abbreviations

AGT: Abnormal glucose tolerance
AUC-ROC: Area under the receiver-operating characteristic curve
GH: Growth hormone
HD–HA: High demand–high adequacy
HD–LA: High demand–low adequacy
1/HISI: Reciprocal of hepatic insulin sensitivity index
IDI: Integrated discrimination improvement
IFG+IGT: Combination of impaired fasting glucose and impaired glucose tolerance
i-IFG: Isolated impaired fasting glucose
i-IGT: Isolated impaired glucose tolerance
LD–HA: Low demand–high adequacy
LD–LA: Low demand–low adequacy
NGT: Normal glucose tolerance
NRI: Net reclassification improvement
RAD: Natural logarithm of ratio between the exponential functions of insulin adequacy and insulin demand
SMA: Standardised major axis
