# Supplementary material for: The relationship of changes in insulin demand and insulin adequacy over the life course
Source: Diabetologia. 2024 Nov 29;68(3):526–36. doi: 10.1007/s00125-024-06328-9 (PMC11832617; doi:10.1007/s00125-024-06328-9)
Supplement: Supplementary file 1 — ESM (PDF 1827 KB) [file 125_2024_6328_MOESM1_ESM.pdf]

Electronic supplementary materials (ESM)

ESM Method 1. Data source and participants

The age range of the lifecourse had been classified by the World Health Organization (WHO) (https://www.who.int/health-topics/adolescent-health#tab=tab_1). Based on the WHO definition, the childhood age was defined as 5-9 years old, adolescence stage as 10-19 years and adulthood stage as 20 years or above. Between 2009 and 2013, offspring of mothers who participated in the Hong Kong field center of the international multi-centre Hyperglycemia and Adverse Pregnancy Outcomes Study[1], were recruited for baseline assessment at 7 years[2] with re-evaluation 4 years after the enrolment (around 11 years old) [3]. In a survey of adolescence obesity, adolescents were recruited from Hong Kong secondary schools in 2003[4, 5]. Using a computer-generated coding system, 53 schools were selected from 477 secondary schools distributed all over Hong Kong with a population of 6.7 million then and 14 schools participated. Adolescents with a mean age of 15.0±1.8 years from each participating school were randomly selected from classes ranging among Form 1-Form 6 (corresponding to Year 7-Year 12 in the United States) with a subgroup undergoing 5-timepoint OGTT. Participants in the adulthood cohort were from the Better Health for Better Hong Kong (BHBHK)- Hong Kong Family Diabetes Study (HKFDS) study. In 1998-2003, the BHBHK-HKFDS cohort[6, 7] was established to investigate metabolic health in young adults with re-evaluation in 2010-2014 (10 years after the enrolment). The BHBHK study recruited members of two leading labor associations in Hong Kong with 236 sub-unions and 450 000 members covering more than 50% of the “grass-root” working population[6]. The HKFDS study recruited first-degree relatives (siblings and parents) of index patients with type 2 diabetes predominantly of young-onset (diagnosed before age of 40 years) attending the Prince of Wales Hospital in 1998-2002, with a response rate of

up to 70%. In this analyses, one adult without diabetes was randomly selected from each family in the HKFDS cohort. We assumed the patients were most likely to be type 2 diabetes, which was the most common type in Chinese population, although there was no specific subtype classification in our study. Exclusion criteria of baseline data for this analysis included: 1) outside the World Health Organization age range for each respective age stage or missing age information; 2) report of vomiting or incomplete 5-timepoint records during OGTT; 3) chronic kidney disease that may affect the OGTT results (eGFR< 60mL/min/1.73m^2); 4) glucose values outside the acceptable range of the HOMA2 calculator (acceptable range for glucose: 3.0-25.0 mmol/L, acceptable range for insulin 20-400 pmol/L); 5) known diagnosis of diabetes.

The study populations at each age group were considered to be representative of each of the age group being examined, as they were recruited from the community from the general Hong Kong population with appropriate epidemiological methods. The children recruited were offspring from pregnant mothers consecutively recruited into the HAPO study, with a few exclusion criteria for the mothers. The adolescent cohort was recruited through a school-based survey that involved random sampling and selection of schools in Hong Kong. The adults were recruited from a community-based epidemiological study of adults from the workforce, together with some additional adults identified through a family study.

Although some subjects were excluded from the analysis, the overall distribution of age, and sex of the included subjects were similar to the distribution of these key parameters in the overall cohort for each age group. In comparing the baseline characteristics of the excluded subjects from those included in the analysis, the male/female distribution were not different, and the age was comparable between the two groups, except

numerically lower age in the adolescents included in the analysis compared to those adolescents excluded. BMI was in general higher among those included in the analysis compared to those excluded from the analysis. Other metabolic parameters were also comparable, except the higher rates of obesity among those included in the analysis compared to those excluded. This was partly due to the restrictions imposed by the HOMA-2 calculator for "acceptable ranges of glucose and insulin", with glucose and insulin levels in general higher among those included in the analysis compared to those excluded.

ESM Method 2. Glucose tolerance definitions

All selected participants underwent a 2-hour OGTT after at least 8 hours of fasting, with a glucose load of 1.75g/kg body weight (maximum 75g) for children and 75g for adults. Blood samples drawn during the OGTT were measured for plasma glucose and insulin at five timepoints (0min, 15min, 30min, 60min and 120min). Glucose tolerance definitions referred to the American Diabetes Association guidelines [8]:

1) Normal glucose tolerance (NGT): fasting plasma blood glucose (FPG) < 5.6 mmol/L and 2-hour PG < 7.8 mmol/L
2) Isolated impaired fasting glucose (i-IFG): FPG 5.6-6.9 mmol/L and 2-hour PG < 7.8 mmol/L
3) Isolated impaired glucose tolerance (i-IGT): FPG < 5.6 mmol/L and 2-hour PG 7.8–11.0 mmol/L
4) Combination of IFG and IGT (IFG+IGT): FPG 5.6-6.9 mmol/L and 2-hour PG 7.8–11.0 mmol/L
5) Diabetes: FPG≥7.0 mmol/L or 2-hour PG≥11.1 mmol/L.

ESM Method 3. Age-sex specific cutoffs of body mass index

We calculated body mass index (BMI) to define overweight/obesity within each age stage. For children and adolescents under 18 years old, we used the age-sex specific cutoffs of BMI aligned with an international standard definition survey, which included data from Hong Kong participants in their sample [9]. For those aged 18 years and older, a BMI cut-off of ≥25kg/m^2 was considered as overweight/obesity.

ESM Method 4. Laboratory measurements

For children, plasma glucose was measured by the hexokinase method, using an automated analyzer (Hitachi 911, Boehringer Mannheim, Mannheim, Germany). Plasma insulin was analysed using an immunoassay analyzer (Immulite 1000 Immunoassay System; Siemens, Munich, Germany). Lipid profiles were measured with enzymatic methods, using a DP Modular Analytics system (Roche Diagnostics, Indianapolis, the United States). For adolescents, plasma glucose was measured by the hexokinase method, using an automated analyzer (Hitachi 911, Boehringer Mannheim, Mannheim, Germany). Total cholesterol, plasma triglycerides, HDL-cholesterol were measured on a Roche Modular Analytics system using standard reagent kits supplied by the manufacturer of the analyser (Roche Diagnostics GmbH, Mannheim, Germany). LDL-cholesterol was calculated using the Friedewald's formula for plasma triglycerides <4.5 mmol/L [10]. For adults, glucose and lipid profiles were measured by appropriate enzymatic methods using a DP analyser (Roche Diagnostics, Hoffmann-La Roche Ltd, Basel, Switzerland).

ESM Table 1. Baseline characteristics of participants along the life course

Variables	Children	Adolescents	Adults	*p*-value
**N**	204	214	605	-
**Age (years old)**	7.0 ± 0.5	15.0 ± 1.8	41.5 ± 9.3	<0.001
**Male (n, %)**	109 (53.4%)	93 (43.5%)	288 (47.6%)	0.122
**BMI (kg/m^2)**	16.42 ± 2.99	20.97 ± 4.44	24.06 ± 3.74	<0.001
**Overweight/Obese (n, %)**	59 (28.9%)	54 (25.2%)	208 (34.4%)	0.032
**SBP (mmHg)**	105.06 ± 9.09	120.48 ± 13.91	118.45 ± 18.58	<0.001
**DBP (mmHg)**	63.89 ± 9.20	73.54 ± 9.98	75.66 ± 11.36	<0.001
**TC (mmol/L)**	4.47 ± 0.71	4.21 ± 0.80	5.15 ± 0.94	<0.001
**TG (mmol/L)**	0.70 (0.60 – 0.90)	0.78 (0.59 – 1.05)	1.06 (0.76 – 1.56)	<0.001
**HDL-c (mmol/L)**	1.65 ± 0.36	1.51 ± 0.31	1.51 ± 0.43	<0.001
**LDL-c (mmol/L)**	2.47 ± 0.59	2.29 ± 0.70	3.06 ± 0.83	<0.001
**GLU0 (mmol/L)**	4.68 ± 0.33	4.78 ± 0.36	4.88 ± 0.51	<0.001
**GLU120(mmol/L)**	5.47 ± 0.89	6.17 ± 1.34	6.20 ± 1.69	<0.001
**INS0 (pmol/L)**	33.90 (25.02 – 48.67)	60.00 (40.92 – 95.75)	49.31 (35.05 – 68.70)	<0.001
**INS120 (pmol/L)**	180.00 (104.40 – 318.60)	503.31 (313.82 – 914.18)	317.26 (203.93 – 483.60)	<0.001
**AGT (n, %)**	7 (3.4%)	34 (15.9%)	133 (22.0%)	<0.001

Descriptive statistics were presented as mean±SD, Median (IQR) or number (%). Abbreviations: N, number; BMI, body mass index; SBP, systolic blood pressure; DBP, diastolic blood pressure; TC, total cholesterol; TG, triglycerides; HDL-c, high-density lipoprotein cholesterol; LDL-c, low-density lipoprotein cholesterol; GLU0, fasting serum glucose at 0 min during oral glucose tolerance test (OGTT); GLU120, serum glucose at 120min during OGTT; INS0, fasting serum insulin at 0 min during OGTT; INS120, serum insulin at 120 min during OGTT; AGT, abnormal glucose tolerance (including impaired fasting glucose tolerance, impaired glucose tolerance, and both impaired fasting glucose tolerance and impaired glucose tolerance at baseline).

ESM Table 2. Insulin sensitivity and insulin secretion of participants during the life course

Variables		Children	Adolescents	Adults	*p*-value
**N**		204	214	605	-
**Fasting phase**	HOMA2-%S	157.60 (111.08 – 212.98)	90.60 (56.62 – 134.38)	109.80 (77.70 – 153.80)	<0.001
	HOMA2-%B[a]	83.30 (65.47 – 106.80)	112.80 (92.40 – 154.12)	97.30 (77.00 – 121.80)	<0.001
**Early phase**	1/HISI	9.73×10^{-7} (6.13×10^{-7} – 1.54×10^{-6})	4.98×10^{-7} (3.45×10^{-7} – 7.96×10^{-7})	8.10×10^{-7} (5.23×10^{-7} – 1.17×10^{-6})	<0.001
	Stumvoll index-1[a]	966.86 (824.66 – 1135.11)	1454.82 (1155.94 – 2081.45)	1080.48 (855.84 – 1430.63)	<0.001
**Late phase**	Matsuda index [a]	7.32 (5.35 – 9.71)	3.31 (2.20 – 4.56)	4.31 (3.08 – 6.05)	<0.001
	Stumvoll index-2[a]	255.02 (220.35 – 295.37)	378.12 (310.35 – 527.89)	290.87 (237.83 – 372.81)	<0.001

Insulin sensitivity in the fasting phase, early phase and late phase were represented by HOMA2-%S, 1/HISI and Matsuda index respectively. Insulin secretion in the fasting phase, early phase and late phase were represented by HOMA2-%B, Stumvoll index-1 and Stumvoll index-2.

Descriptive statistics were presented by median (IQR). "a" Statistical differences were observed between each two group (children vs adolescents, children vs adults, and adults vs adolescents) using post hoc tests. Abbreviations: N, number.

ESM Table 3. Insulin sensitivity and insulin secretion stratified by glucose tolerance and body weight

Variables		Children	Adolescents	Adults	*p*-value
**NGT**					
**N**		197	180	472	-
**Fasting phase**	HOMA2-%S[a]	158.50 (111.20 – 212.60)	89.95 (56.45 – 130.32)	112.80 (81.77 – 155.90)	<0.001
	HOMA2-%B[a]	84.00 (66.10 –106.60)	114.65 (94.07 –157.30)	99.50 (80.38 –121.90)	<0.001
**Early phase**	1/HISI[a]	9.70×10^{-7} (5.99×10^{-7} – 1.52×10^{-6})	4.90×10^{-7} (3.46×10^{-7} – 7.89×10^{-7})	8.16×10^{-7} (5.41×10^{-7} – 1.21×10^{-6})	<0.001
	Stumvoll index-1[a]	970.06 (832.68 – 1139.88)	1504.55 (1222.41 – 2095.90)	1124.37 (918.59 – 1445.83)	<0.001
**Late phase**	Matsuda index[a]	7.27 (5.36 – 9.93)	3.40 (2.24 – 4.63)	4.68 (3.34 – 6.36)	<0.001
	Stumvoll index-2[a]	256.42 (223.22 – 296.62)	391.13 (323.28 – 533.68)	297.54 (248.74 – 373.32)	<0.001
**Non-overweight/Non-obese**					
**N**		145	160	397	
**Fasting phase**	HOMA2-%S[a]	169.40 (117.20 – 223.90)	101.55 (67.35 – 152.02)	125.70 (92.90 – 166.10)	<0.001
	HOMA2-%B[a]	77.90 (64.20 – 100.50)	106.80 (83.35 – 129.55)	92.00 (74.90 – 112.50)	<0.001
**Early phase**	1/HISI[a]	1.15×10^{-6} (7.80×10^{-7} – 1.63×10^{-6})	5.78×10^{-7} (3.98×10^{-7} – 9.09×10^{-7})	9.14×10^{-7} (6.44×10^{-7} – 1.28×10^{-6})	<0.001

	Stumvoll index-1[a]	905.07 (759.53 – 1063.19)	1304.38 (1113.86 – 1779.40)	1034.32 (848.36 – 1332.66)	<0.001
**Late phase**	Matsuda index[a]	8.07 (6.14 – 10.79)	3.65 (2.72 – 5.18)	5.08 (3.64 – 6.67)	<0.001
	Stumvoll index-2[a]	239.77 (208.09 – 281.71)	339.32 (294.96 – 459.17)	277.33 (232.77 – 348.16)	<0.001
**NGT + Non-overweight/Non-obese**					
**N**		140	136	330	
**Fasting phase**	HOMA2-%S[a]	171.65 (117.27 – 222.48)	100.80 (67.35 – 150.85)	126.90 (94.50 – 168.82)	<0.001
	HOMA2-%B[a]	79.85 (64.57 – 100.80)	107.25 (83.97 – 132.62)	93.70 (76.55 – 113.72)	<0.001
**Early phase**	1/HISI[a]	1.14×10^{-6} (7.79×10^{-7} – 1.59×10^{-6})	5.57×10^{-7} (3.85×10^{-7} – 9.11×10^{-7})	9.26×10^{-7} (6.45×10^{-7} – 1.30×10^{-6})	<0.001
	Stumvoll index-1[a]	909.38 (788.66 – 1067.40)	1341.01 (1125.20 – 1887.80)	1068.03 (893.71 – 1354.38)	<0.001
**Late phase**	Matsuda index[a]	8.07 (6.12 – 10.89)	3.85 (2.77 – 5.22)	5.30 (3.82 – 6.92)	<0.001
	Stumvoll index-2[a]	241.29 (210.78 – 282.95)	351.29 (297.60 – 482.08)	285.88 (244.45 – 352.51)	<0.001

Insulin sensitivity in the fasting phase, early phase and late phase were represented by HOMA2-%S, 1/HISI and Matsuda index respectively. Insulin secretion in the fasting phase, early phase and late phase were represented by HOMA2-%B, Stumvoll index-1 and Stumvoll index-2.

Descriptive statistics were presented by median (IQR). "a" Statistical differences were observed between each two group (children vs adolescents, children vs adults, and adults vs adolescents) using post hoc tests. Abbreviations: N, number; NGT, normal glucose tolerance.

ESM Table 4. RAD values of the life course cohort during 3 phases of the OGTT stratified by glucose tolerance and body weight

Variables	Children	Adolescents	Adults	*p*-value
**NGT**				
**N**	197	180	472	-
**Fasting phase RAD**[a]	0.63 (0.06 – 1.09)	-0.26 (-0.99 – 0.31)	0.11 (-0.39 – 0.58)	<0.001
**Early phase RAD**[a]	0.38 (-0.25 – 0.97)	-0.46 (-0.93 – 0.17)	0.16 (-0.38 – 0.67)	<0.001
**Late phase RAD**[a]	0.66 (0.26 – 1.08)	-0.31 (-0.84 – 0.10)	0.08 (-0.36 – 0.45)	<0.001
**Non-overweight/Non-obese**				
**N**	145	160	397	-
**Fasting phase RAD**[a]	0.73 (0.14 – 1.15)	-0.09 (-0.70 – 0.53)	0.26 (-0.20 – 0.70)	<0.001
**Early phase RAD**[a]	0.62 (0.04 – 1.09)	-0.30 (-0.80 – 0.30)	0.31 (-0.14 – 0.76)	<0.001
**Late phase RAD**[a]	0.78 (0.43 – 1.17)	-0.21 (-0.61 – 0.27)	0.16 (-0.23 – 0.50)	<0.001
**NGT + Non-overweight/Non-obese**				
**N**	140	136	330	-
**Fasting phase RAD**[a]	0.74 (0.14 – 1.15)	-0.10 (-0.70 – 0.51)	0.26 (-0.18 – 0.70)	<0.001
**Early phase RAD**[a]	0.61 (0.03 – 1.09)	-0.31 (-0.81 – 0.34)	0.34 (-0.15 – 0.81)	<0.001
**Late phase RAD**[a]	0.82 (0.43 – 1.18)	-0.16 (-0.58 – 0.28)	0.24 (-0.16 – 0.58)	<0.001

RAD was calculated using the following formula, based on insulin demand and insulin adequacy during different OGTT phases:

$$\text{RAD} = \log_e\left(\frac{\exp(insulin\ adequacy)}{\exp(insulin\ demand)}\right)$$

Descriptive statistics were presented by median (IQR). "a" Statistical differences were observed between each two group (children vs adolescents, children vs adults, and adults vs adolescents) using post hoc tests. Abbreviations: N, number; RAD, natural logarithm of ratio between the exponential functions of insulin adequacy and demand.

ESM Table 5. Baseline characteristics in participants with and without follow-up records

Variables	Children			Adults		
	With follow-up records	Without records	*p*-value	With follow-up records	Without records	*p*-value
**N**	89	115	-	233	372	-
**Age (years old)**	7.1±0.5	6.9±0.4	0.010	40.9±11.7	41.9±7.4	0.187
**Male (n, %)**	48(54.5%)	61(52.6%)	0.892	116(49.8%)	172(46.2%)	0.443
**BMI (kg/m²)**	16.88±3.34	16.07±2.65	0.054	23.65±3.75	24.32±3.72	0.032
**Overweight/Obese (n, %)**	30(34.1%)	29(25.0%)	0.207	76(32.6%)	132(35.5%)	0.526
**SBP (mmHg)**	106.09±9.60	104.28±8.64	0.160	120.35±21.03	117.26±16.79	0.046
**DBP (mmHg)**	65.59±9.40	62.60±8.87	0.021	76.48±12.29	75.14±10.73	0.159
**TC (mmol/L)**	4.42±0.66	4.51±0.75	0.388	5.11±0.89	5.17±0.96	0.455
**TG (mmol/L)**	0.70 (0.50 – 0.90)	0.70 (0.60 – 1.00)	0.353	1.05 (0.72 – 1.54)	1.07 (0.77 – 1.59)	0.271
**HDL-c (mmol/L)**	1.63±0.34	1.66±0.38	0.453	1.48±0.39	1.53±0.45	0.167
**LDL-c (mmol/L)**	2.46±0.56	2.48±0.61	0.776	3.05±0.79	3.07±0.86	0.704
**GLU0 (mmol/L)**	4.65±0.35	4.70±0.31	0.299	4.84±0.53	4.91±0.49	0.101
**GLU120 (mmol/L)**	5.46±0.96	5.48±0.85	0.896	6.24±1.68	6.18±1.70	0.653
**INS0 (pmol/L)**	34.38 (25.80 – 45.60)	33.48 (24.90 – 49.50)	0.756	49.02 (33.42 – 65.70)	49.56 (36.54 – 70.44)	0.277
**INS120 (pmol/L)**	188.10 (100.92 – 319.62)	171.90 (106.38 – 313.20)	0.910	325.92 (210.12 – 484.80)	311.34 (203.58 – 482.52)	0.660
**AGT (n, %)**	3(3.41%)	4(3.45%)	0.971	52(22.3%)	81(21.8%)	0.977

Descriptive statistics were presented as mean±SD, Median (IQR) or number (%). Abbreviations: N, number; BMI, body mass index; SBP, systolic blood pressure; DBP, diastolic blood pressure; TC, total cholesterol; TG, triglycerides; HDL-c, high-density lipoprotein cholesterol; LDL-c, low-density lipoprotein cholesterol; GLU0, fasting

serum glucose at 0 min during oral glucose tolerance test (OGTT); GLU120, serum glucose at 120min during OGTT; INS0, fasting serum insulin at 0 min during OGTT; INS120, serum insulin at 120 min during OGTT; AGT, abnormal glucose tolerance (including impaired fasting glucose tolerance, impaired glucose tolerance, and both impaired fasting glucose tolerance and impaired glucose tolerance at baseline).

ESM Table 6. Performance of RAD models in evaluating the risk of AGT by glycaemic trajectories in the adult group

Variables	NGT at baseline - NGT at follow-up	NGT at baseline - AGT at follow-up	*p*-value
**N (%)**	214 (93.4%)	15 (6.6%)	-
**Fasting RAD**	0.16 (-0.37 – 0.54)	-0.11 (-1.36 – 0.34)	0.146
**Early phase RAD**	0.22 (-0.27 – 0.62)	-0.13 (-1.08 – 0.02)	0.032
**Late phase RAD**	0.10 (-0.30 – 0.44)	-0.26 (-1.10 – 0.09)	0.021

Descriptive statistics are presented by median (IQR). Abbreviations: N, number; RAD, natural logarithm of ratio between the exponential functions of insulin adequacy and demand; NGT normal glucose tolerance; AGT, abnormal glucose tolerance.

ESM Table 7. Performance of models in evaluating the risk of diabetes in adults

Methods	Fasting phase	Early phase	Late phase
**Insulin demand–adequacy method[a]**			
OR (95%CI)	1.53 (0.90, 2.61)	1.59 (0.90, 2.78)	3.51 (2.04, 6.11)
AUC-ROC (95%CI)	0.577 (0.502, 0.651)	0.571 (0.492, 0.649)	0.664 (0.588, 0.740)
NRI (95%CI)	0.213 (-0.048, 0.474)	0.201 (-0.048, 0.449)	0.601 (0.346, 0.856)
IDI (95%CI)	0.007 (-0.002, 0.016)	0.008 (-0.002, 0.018)	0.061 (0.035, 0.088)
**RAD method[b]**			
OR (95%CI)	1.84 (1.04, 3.21)	2.21 (1.21, 3.99)	3.01 (1.72, 5.24)
AUC-ROC (95%CI)	0.596 (0.521, 0.672)	0.580 (0.498, 0.662)	0.633 (0.566, 0.712)
NRI (95%CI)	0.249 (0.002, 0.496)	0.286 (0.049, 0.523)	0.464 (0.211, 0.717)
IDI (95%CI)	0.012 (0.001, 0.025)	0.021 (0.004, 0.038)	0.045 (0.021, 0.070)

The base model was adjusted for age and sex (AUC-ROC=0.541). The insulin demand–adequacy models and RAD models were also adjusted for age and sex. [a] In the insulin demand–adequacy method, participants were divided into 4 groups stratified by the median values of insulin demand and insulin adequacy: LD–HA, HD–HA, HD–LA and LD–LA. The combined group of LD–LA+HD–HA+LD–HA were taken as the reference group for comparisons to the at-risk group (the HD–LA group). [b] In the RAD method, participants were divided into 4 groups according to quartiles of RAD values, with individuals of RAD values above the 25th percentile as the reference group for comparisons to the at-risk group (RAD values below the 25th percentile). Abbreviations: RAD, natural logarithm of ratio between the exponential functions of insulin adequacy and demand; OR, odds ratio; AUC-ROC, area under the receiver-operating characteristic (ROC) curve; NRI, net reclassification improvement index; IDI, integrated discrimination improvement index; low demand–high adequacy (LD–HA); high demand–high adequacy (HD–HA); high demand–low adequacy (HD–LA) and low demand–low adequacy (LD–LA)

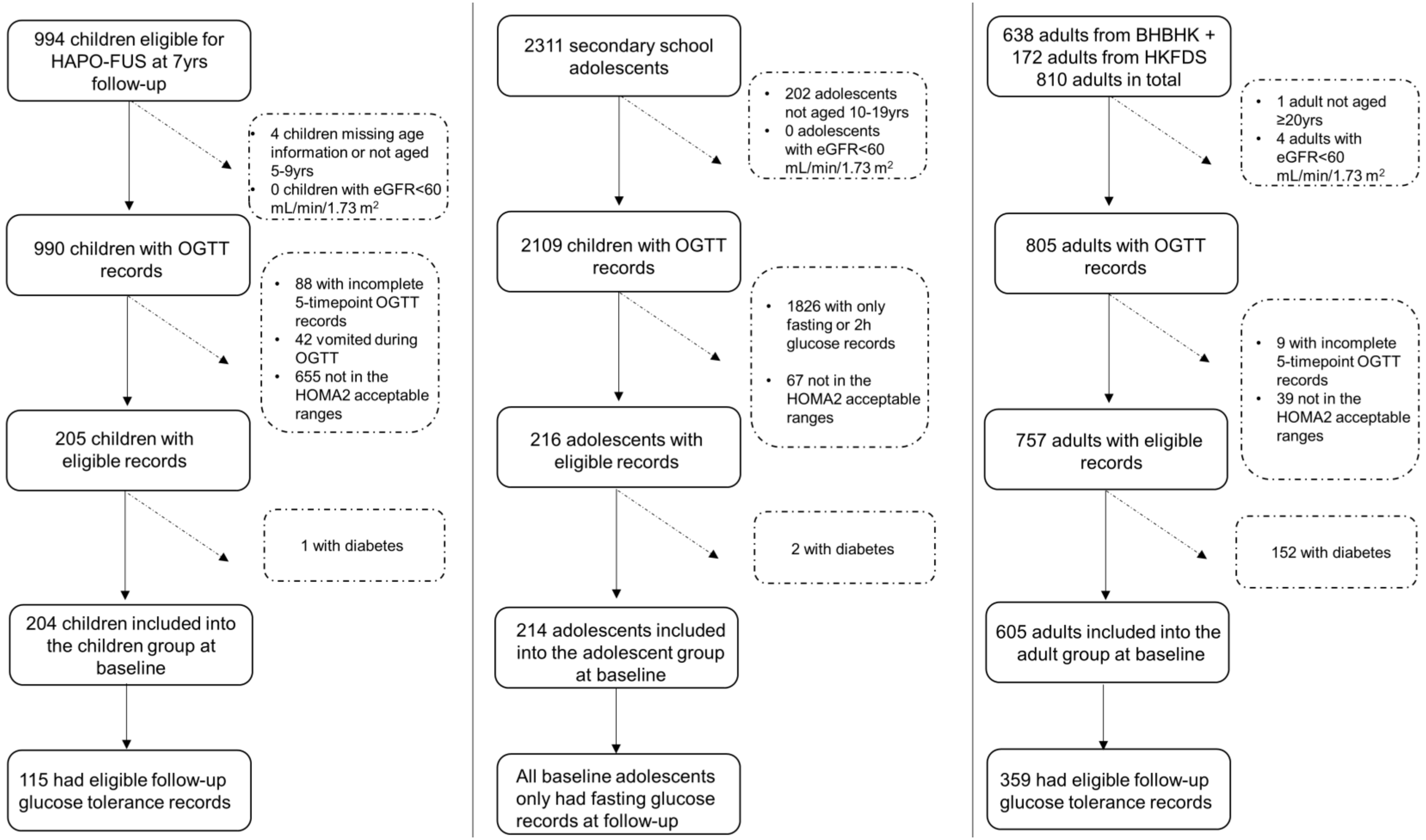

ESM Fig 1. Flowchart of participants at baseline and eligible for follow-up glucose tolerance records

Acceptable range for glucose in HOMA2 calculator: 3.0-25.0 mmol/L. Acceptable range for insulin in HOMA2 calculator: 20-400 pmol/L

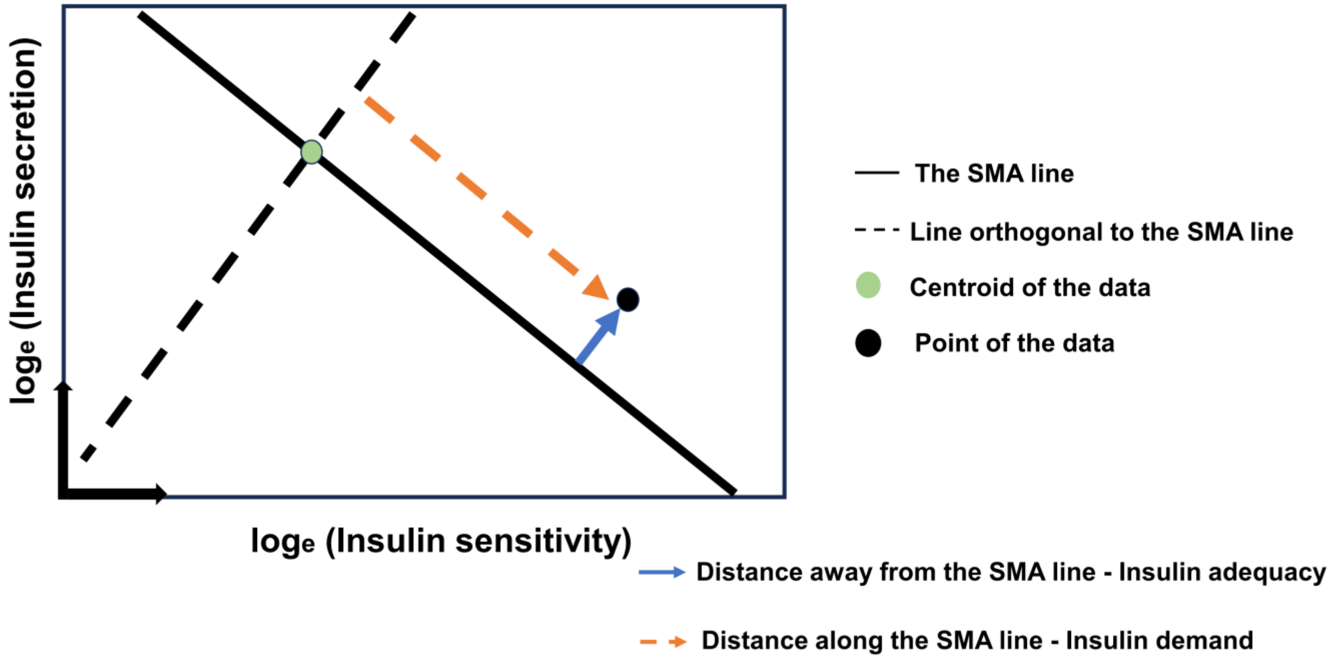

ESM Fig 2. Schematic diagram of distances away from the SMA line and distances along the SMA line

The SMA line is a fitted line plotted by the SMA regression of all points in the data according to the formula: $\log_e$ (Insulin secretion) = a + b * $\log_e$ (Insulin sensitivity). The orthogonal line is a perpendicular line to the SMA line which goes through the centroid of the data. The green dot represents centroid of the data, which is set as (m, n) in the coordinate system which corresponds to mean of $\log_e$ (Insulin sensitivity) and mean of $\log_e$ (Insulin secretion) numerically. The black point represents a point of the data (x, y). Distances away from the SMA line are calculated using the formula $d1 = \frac{y-(a+b*x)}{\sqrt{1+b^2}}$; distances along the SMA line are calculated using the formula $d2 = \frac{\frac{1}{b}*x+y-(n+\frac{1}{b}*m)}{\sqrt{1+\frac{1}{b^2}}}$.

Distances can be negative and positive, taking the same directions as the residuals. Distance along the SMA line represents insulin demand and distance away from the SMA line represents insulin adequacy.

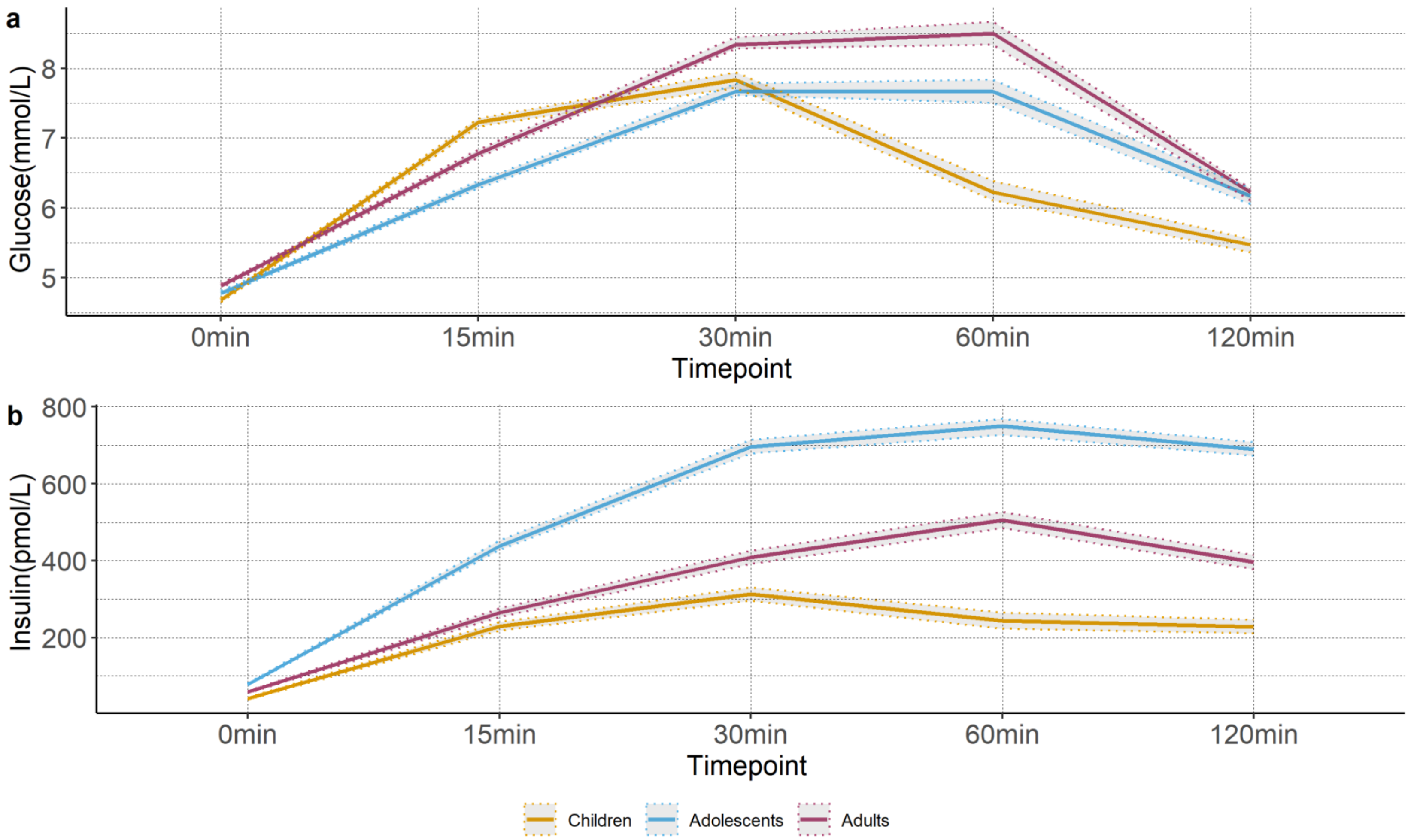

ESM Fig 3. Dynamic glucose and insulin changes of OGTT in children, adolescents and adults along the lifecourse

a: Dynamic glucose changes during OGTT along the lifecourse; b: Dynamic insulin changes during OGTT along the life course. Solid lines in the middle showed the mean value of glucose or insulin; dotted lines on either side represent the 95% CI of glucose or insulin values. Abbreviations: OGTT, oral glucose tolerance test; 95% CI, 95% confidence interval.

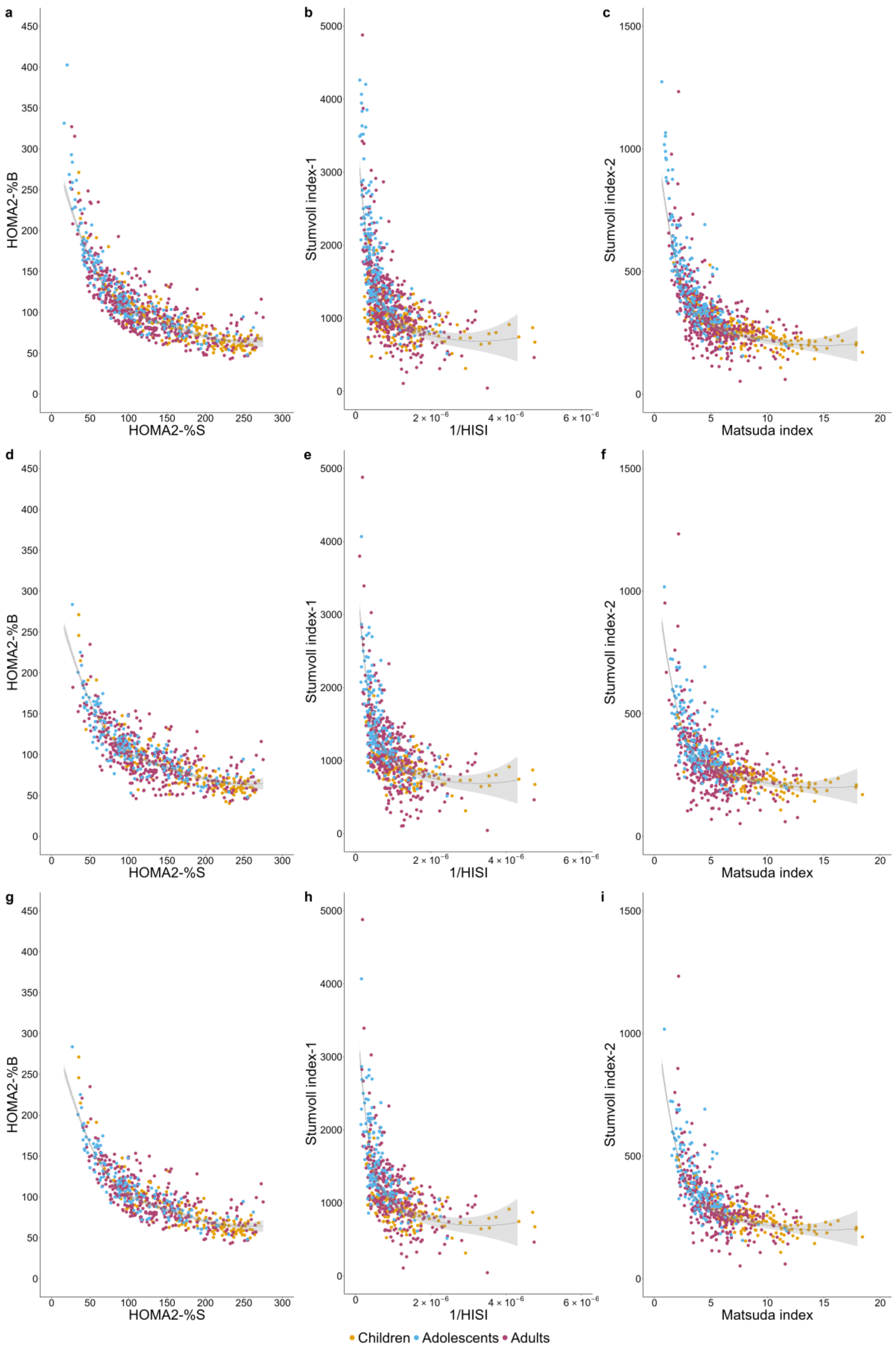
a
b
c
d
e
f
g
h
i
HOMA2-%B
HOMA2-%S
Stumvoll index-1
1/HISI
Stumvoll index-2
Matsuda index
Children
Adolescents
Adults

ESM Fig 4. Insulin sensitivity (x axis) and secretion indices (y axis) over the life course (in children, adolescents and adults) during the fasting, early and late phase of the OGTTs, stratified by glucose tolerance and body weight

Fasting phases insulin sensitivity-secretion indices were presented by HOMA2-%S and HOMA2-%B; Early phase insulin sensitivity-secretion indices were presented by 1/HISI and Stumvoll index-1; Late phase insulin sensitivity-secretion indices were presented by Matsuda index and Stumvoll index-2. (a)-(c): NGT individuals; (d)-(f): Non-overweight/Non-obese individuals; (g)-(i): NGT + Non-overweight/Non-obese individuals. The dotted curve showed a fitted curvilinear line of the insulin sensitivity and insulin secretion and the grey shaded area showed the 95% CI of the curvilinear line Abbreviations: NGT, normal glucose tolerance.

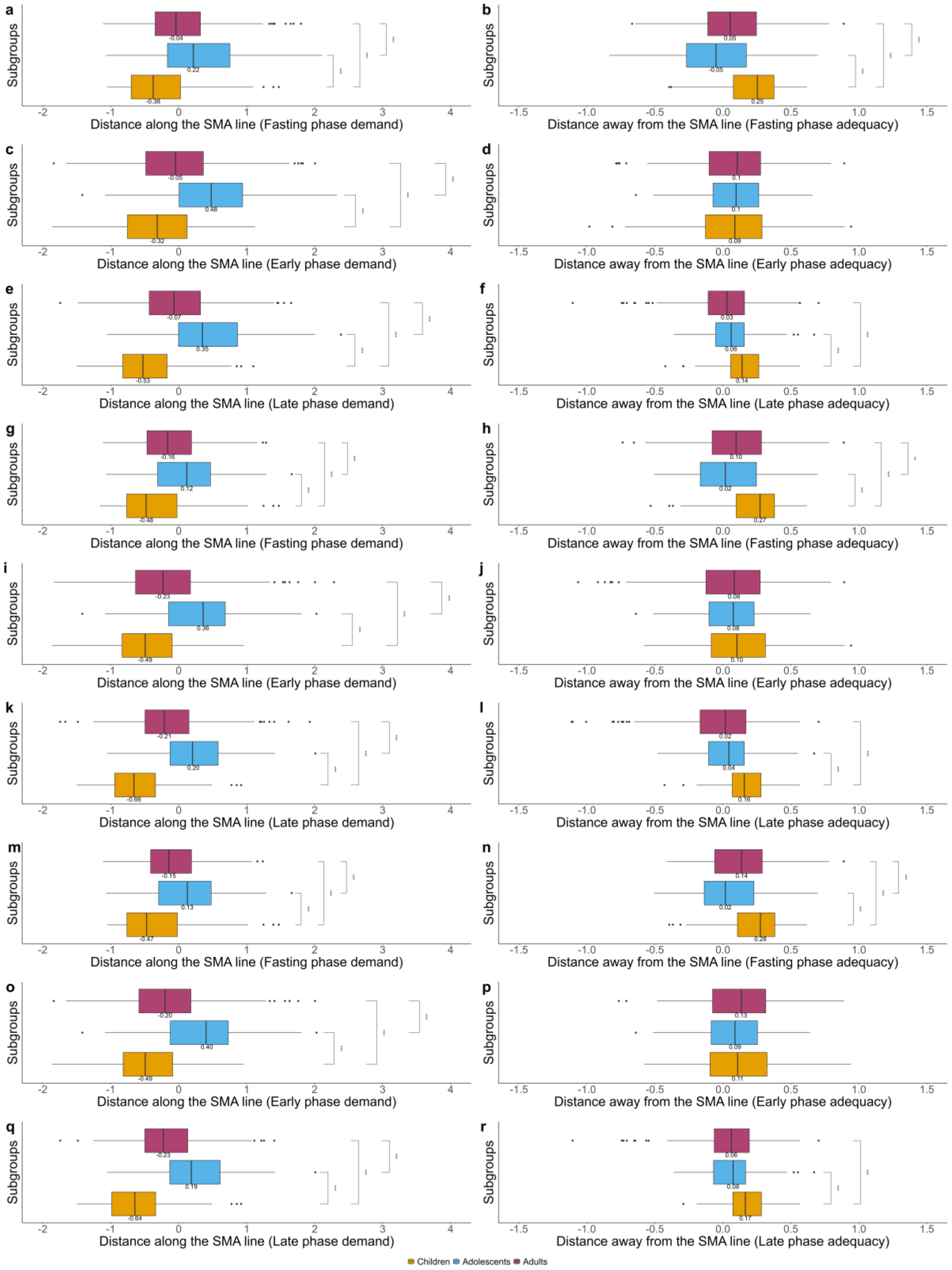
a
Subgroups
-0.04
0.22
-0.38
-2 -1 0 1 2 3 4
Distance along the SMA line (Fasting phase demand)
b
Subgroups
0.05
-0.05
0.25
-1.5 -1.0 -0.5 0.0 0.5 1.0 1.5
Distance away from the SMA line (Fasting phase adequacy)
c
Subgroups
-0.05
0.48
-0.32
Distance along the SMA line (Early phase demand)
d
Subgroups
0.1
0.1
0.09
Distance away from the SMA line (Early phase adequacy)
e
Subgroups
-0.07
0.35
-0.53
Distance along the SMA line (Late phase demand)
f
Subgroups
0.03
0.06
0.14
Distance away from the SMA line (Late phase adequacy)
g
Subgroups
-0.16
0.12
-0.48
Distance along the SMA line (Fasting phase demand)
h
Subgroups
0.10
0.02
0.27
Distance away from the SMA line (Fasting phase adequacy)
i
Subgroups
-0.23
0.36
-0.49
Distance along the SMA line (Early phase demand)
j
Subgroups
0.08
0.08
0.10
Distance away from the SMA line (Early phase adequacy)
k
Subgroups
-0.21
0.20
-0.66
Distance along the SMA line (Late phase demand)
l
Subgroups
0.02
0.04
0.16
Distance away from the SMA line (Late phase adequacy)
m
Subgroups
-0.15
0.13
-0.47
Distance along the SMA line (Fasting phase demand)
n
Subgroups
0.14
0.02
0.28
Distance away from the SMA line (Fasting phase adequacy)
o
Subgroups
-0.20
0.40
-0.49
Distance along the SMA line (Early phase demand)
p
Subgroups
0.13
0.09
0.11
Distance away from the SMA line (Early phase adequacy)
q
Subgroups
-0.23
0.19
-0.64
Distance along the SMA line (Late phase demand)
r
Subgroups
0.06
0.08
0.17
Distance away from the SMA line (Late phase adequacy)
Children Adolescents Adults

ESM Fig 5. Insulin demand and insulin adequacy over the life course (in children, adolescents and adults), stratified by glucose tolerance and body weight

The insulin demand and adequacy were derived from insulin sensitivity and secretion indices in each phase using the standardized major axis regression method. Insulin sensitivity in the fasting phase, early phase and late phase were represented by HOMA2-%S, 1/HISI and Matsuda index respectively. Insulin secretion in the fasting phase, early phase and late phase were represented by HOMA2-%B, Stumvoll index-1 and Stumvoll index-2 respectively. (a)-(f): NGT individuals; (g)-(l): Non-overweight/Non-obese individuals; (m)-(r): NGT + Non-overweight/Non-obese individuals. Median values were shown in the box plots in each group. Convention for symbols indicated statistical significance: Convention for symbols indicated statistical significance: $^{**}p< 0.01$; $^{***}p< 0.001$. Abbreviations: NGT, normal glucose tolerance.

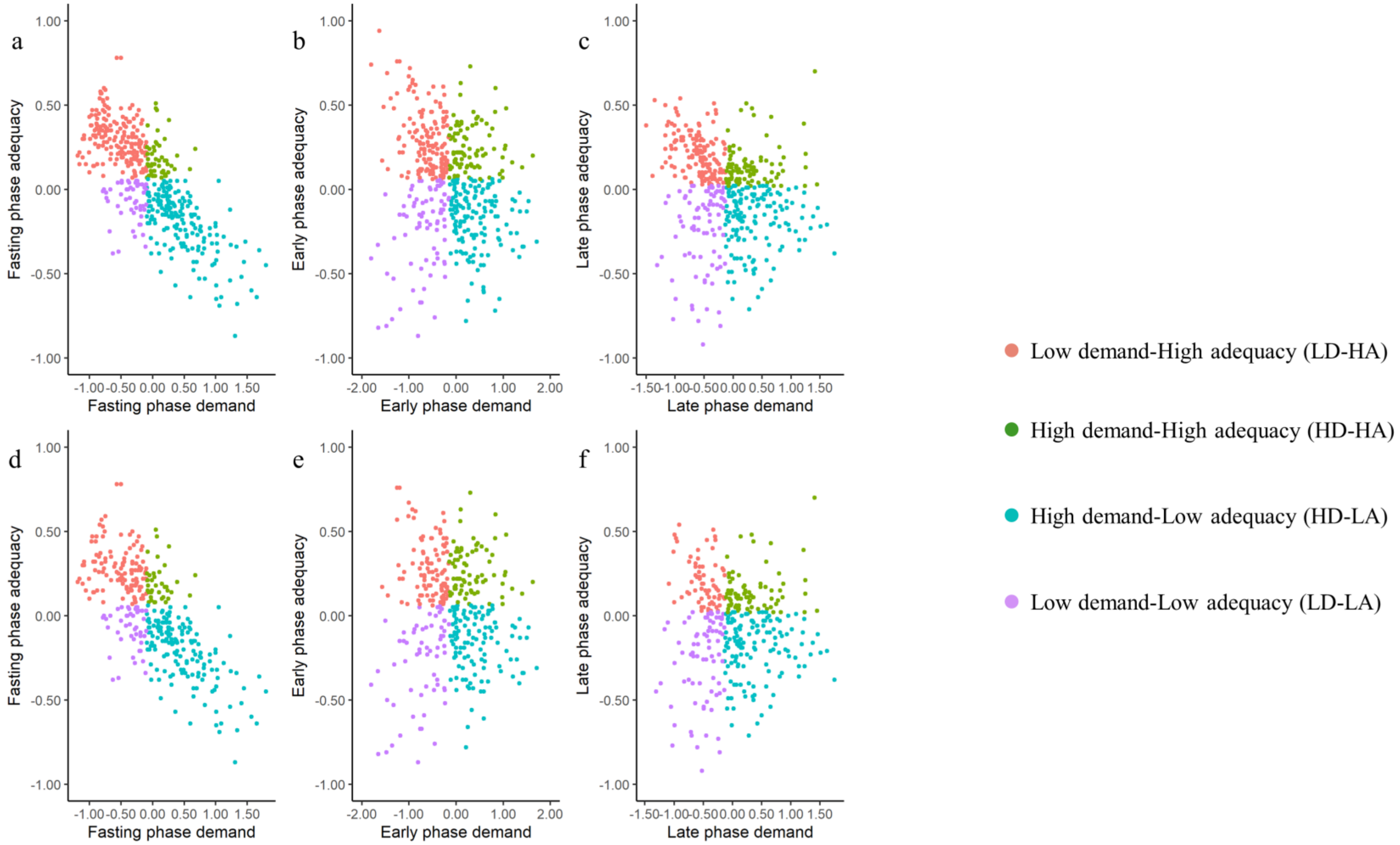

ESM Figure 6 Insulin demand-adequacy distributions of participants

Participants were divided into 4 groups: LD-HA, HD-HA, HD-LA, LD-LA. Abbreviations: low demand-high adequacy (LD-HA), high demand-high adequacy (HD-HA), high demand-low adequacy (HD-LA) and low demand-low adequacy (LD-LA) stratified by the median values of demand and adequacy respectively (a)-(c): children and adults; (d)-(f): adults only.

References

[1] Metzger BE, Lowe LP, Dyer AR, et al. (2008) Hyperglycemia and adverse pregnancy outcomes. N Engl J Med 358(19): 1991-2002. https://doi.org/10.1056/NEJMoa0707943

[2] Tam WH, Ma RCW, Ozaki R, et al. (2017) In Utero Exposure to Maternal Hyperglycemia Increases Childhood Cardiometabolic Risk in Offspring. Diabetes Care 40(5): 679-686. https://doi.org/10.2337/dc16-2397

[3] Lowe WL, Jr., Scholtens DM, Kuang A, et al. (2019) Hyperglycemia and Adverse Pregnancy Outcome Follow-up Study (HAPO FUS): Maternal Gestational Diabetes Mellitus and Childhood Glucose Metabolism. Diabetes Care 42(3): 372-380. https://doi.org/10.2337/dc18-1646

[4] Ozaki R, Qiao Q, Wong GW, et al. (2007) Overweight, family history of diabetes and attending schools of lower academic grading are independent predictors for metabolic syndrome in Hong Kong Chinese adolescents. Arch Dis Child 92(3): 224-228. https://doi.org/10.1136/adc.2006.100453

[5] Kong AP, Choi KC, Ko GT, et al. (2008) Associations of overweight with insulin resistance, beta-cell function and inflammatory markers in Chinese adolescents. Pediatr Diabetes 9(5): 488-495. https://doi.org/10.1111/j.1399-5448.2008.00410.x

[6] Ko GT, Chan JC, Chan AW, et al. (2007) Association between sleeping hours, working hours and obesity in Hong Kong Chinese: the 'better health for better Hong Kong' health promotion campaign. Int J Obes (Lond) 31(2): 254-260. https://doi.org/10.1038/sj.ijo.0803389

[7] Zhang Y, Luk AOY, Chow E, et al. (2017) High risk of conversion to diabetes in first-degree relatives of individuals with young-onset type 2 diabetes: a 12-year follow-up analysis. Diabet Med 34(12): 1701-1709. https://doi.org/10.1111/dme.13516

[8] ElSayed NA, Aleppo G, Aroda VR, et al. (2023) 2. Classification and Diagnosis of Diabetes: Standards of Care in Diabetes-2023. Diabetes Care 46(Suppl 1): S19-s40. https://doi.org/10.2337/dc23-S002

[9] Cole TJ, Bellizzi MC, Flegal KM, et al. (2000) Establishing a standard definition for child overweight and obesity worldwide: international survey. Bmj: 1240-1243. https://doi.org/10.1136/bmj.320.7244.1240

[10] Friedewald WT, Levy RI, Fredrickson DS (1972) Estimation of the concentration of low-density lipoprotein cholesterol in plasma, without use of the preparative ultracentrifuge. Clin Chem 18(6): 499-502
